# Weed responses to fallow management in Pacific Northwest dryland cropping systems

**DOI:** 10.1371/journal.pone.0204200

**Published:** 2018-09-20

**Authors:** Carolina San Martín, Dan S. Long, Jennifer A. Gourlie, Judit Barroso

**Affiliations:** 1 Department of Crop and Soil Science, Columbia Basin Agricultural Research Center (CBARC), Oregon State University, Adams, OR, United States of America; 2 USDA-ARS Soil and Water Conservation Research Unit, Adams, OR, United States of America; Instituto Agricultura Sostenible, SPAIN

## Abstract

A two-year rotation of summer fallow (SF)/winter wheat (WW) is the most common cropping system in low precipitation areas of the U.S. Pacific Northwest (PNW). In SF, multiple tillage operations are used to manage weeds and maximize soil water storage and potential WW yield. Reduced tillage fallow (RTF) is an alternative to SF that leaves >30% of the previous crop’s residue on the surface. A four-year (2014–18) field study was conducted to evaluate the influence of SF and RTF on weed species density, cover and composition in dryland WW; determine if changes in these weed infestation attributes have any influence on crop density and yield; and evaluate economic costs of each type of fallow management. The experimental design was randomized complete block with four replications where each phase of SF/WW and RTF/WW rotations was present every year. Individual plots of WW were divided into a weedy sub-plot with no weed control, general area with chemical weed control, and weed-free sub-plot where weeds were manually removed. Infestations of annual grass and other weeds in weedy sub-plots increased throughout the study. Grass weed cover, consisting mainly of downy brome (*Bromus tectorum* L.), and total weed cover were significantly lower in WW following RTF than following SF in all years except 2018. Densities of grass and total weeds were similar in both fallow managements indicating that weed plants were larger in WW following SF than following RTF due to earlier or faster emergence. Grass cover differences were not found in general areas likely because of a reduced seedbank. When weeds were present, mean yield of WW was higher following RTF than SF indicating that weeds were less competitive in RTF. Reduced tillage fallow could improve weed management in fallow/WW cropping systems of the PNW compared to SF/WW, particularly if the most problematic species are grasses.

## Introduction

The most common cropping system in the winter wheat (WW, *Triticum aestivum* L.) producing region of the U.S. Pacific Northwest is WW followed by one year of fallow [1,2]. The fallow year is used to recharge soil moisture [3,4] as needed to maximize potential WW yield in the following year [5,6]. Conventional summer fallow (SF) involves the use of frequent tillage (e.g., disk harrowing, rod weeding [7]) to control weeds and create a dust mulch that resists evaporation [3,7,8]. Unfortunately, SF is prone to wind erosion that reduces soil productivity and produces dust emissions that impairs air quality and threatens human health [4,9]. To allay concerns about wind erosion, growers are interested in adopting conservation tillage practices such as minimum tillage and no-till [1]. Conservation tillage can be less expensive than SF because of savings of fuel, machinery, labor and time [10,11]. However, high herbicide costs in some years may favor SF [11].

No-till practices involving chemical fallow (CF, controlling weeds in fallow fields with chemicals) have been used successfully in the Great Plains where a summer precipitation pattern helps sustain crops through the hot summer season [12,13]. However, summers are hot and dry in the semiarid PNW and the absence of tillage that otherwise would interrupt capillary flow may cause severe evaporative loss of soil moisture [14,15]. In general, WW yields tend to be higher in rotation with SF than with CF [6,8] because growers can seed WW earlier into SF than into CF [16,17].

Conservation tillage practices minimize soil disturbance, leave vegetative residue on the surface, and rotate primary crop with other crops [18]. Reduced tillage fallow (RTF) involves a single tillage operation with a minimum disturbance implement such as V-blade sweep. Wind erosion is reduced by retaining surface residue and soil clods that increase surface roughness [4,19]. Schillinger [1] showed that crop yield increased following RTF compared to CF. Cardina et al. [20] found that CF increased weed seedbank density, due to lack of seed burial, compared with tillage-based systems.

Changes in tillage regime may cause floristic inversion due to changes in the seedbank [21–23]. Incorporating seeds deeper into the soil with tillage might favor conditions for an increase of seedbank [23,24], but burying seeds might also avoid seed germination [25] and favor seed deterioration [26]. Under no-till and RTF, seeds are more likely to remain near the soil surface [24] where they are susceptible to insect and bird predation in the summer periods [27]. Germination of some species is favored when seeds are near the soil surface and thus no-till and RTF will increase seedling emergence compared with conventional tillage. However, Mohler [24] observed that emergence increased in the first year followed by reduction in later years due to depletion of the seed surface fraction by the action of herbicides and shallow cultivation. Moreover, crop residues associated with no-till and RTF may suppress weeds by blocking sunlight and reducing physical space for seedling emergence [28–30].

Common weeds in the WW phase of the fallow / WW system are winter annual grasses: downy brome and feral rye (*Secale cereale* L.); winter annual broadleaves: prickly lettuce (*Lactuca serriola* L.), tumble mustard (*Sisymbrium altissimum* L.), and flixweed (*Descurainia sophia* (L.) Webb. Ex Prantl); and summer annual broadleaves: Russian thistle (*Salsola tragus* L.), lambsquarters (*Chenopodium* ssp.), tumble pigweed (*Amaranthus albus* L.), fiddleneck (*Amsinckia intermedia* Fisch. & C.A. Mey), horseweed (*Conyza canadiensis* (L.) Cronq.), and prostrate knotweed (*Polygonum aviculare* L) [3]. Horseweed and prickly lettuce have increased in CF in the region [3]. Lambsquarters has increased in tilled systems more than in no-till [20]. Russian thistle has decreased in no-till systems [3]. In general, annual wind-disseminated weeds and grasses tend to occur in reduced and no-till systems whereas non-wind-disseminated weeds are more frequent in tillage systems [31,32]. No-till may favor weeds whose seeds concentrate in the soil surface and are normally not a problem in tillage systems [33,34].

Downy brome, which is one of the most problematic species in WW in the PNW region [3], germinates mainly in fall with first rains and to a lesser extent in early spring [35] to take advantage of moisture and nutrients in advance of crop growth. Similar morphology (life cycle mimicry) to wheat and limited chemical control options help proliferate this weed. In eastern Washington, downy brome infestations of 54 and 500 plants m^-2^ caused 28% to 100% reduction in WW yield [36]. Lyon and Baltensperger [37] found that two-year crop rotations were effective in controlling downy brome if plants were not allowed to produce seeds in the fallow periods. Wicks [38] showed that moldboard plowing effectively controlled this weed species during fallow owing to deep seed burial. Downy brome control might be improved in no-till cropping systems with the help of occasional tillage [39,40].

Fallow management is an important determinant of weed infestations in the following cropping year. Therefore, amount of crop residue and degree of soil disturbance will influence the composition and density of weeds. The objectives of this research were to: (1) evaluate the influence of SF and RTF on density, cover and composition of downy brome and other weed species in dryland WW; (2) determine if changes in these weed infestation attributes influence crop density and yield; and (3) evaluate economic costs of each type of fallow management.

## Materials and methods

### Site description and history

**Fig 1:**
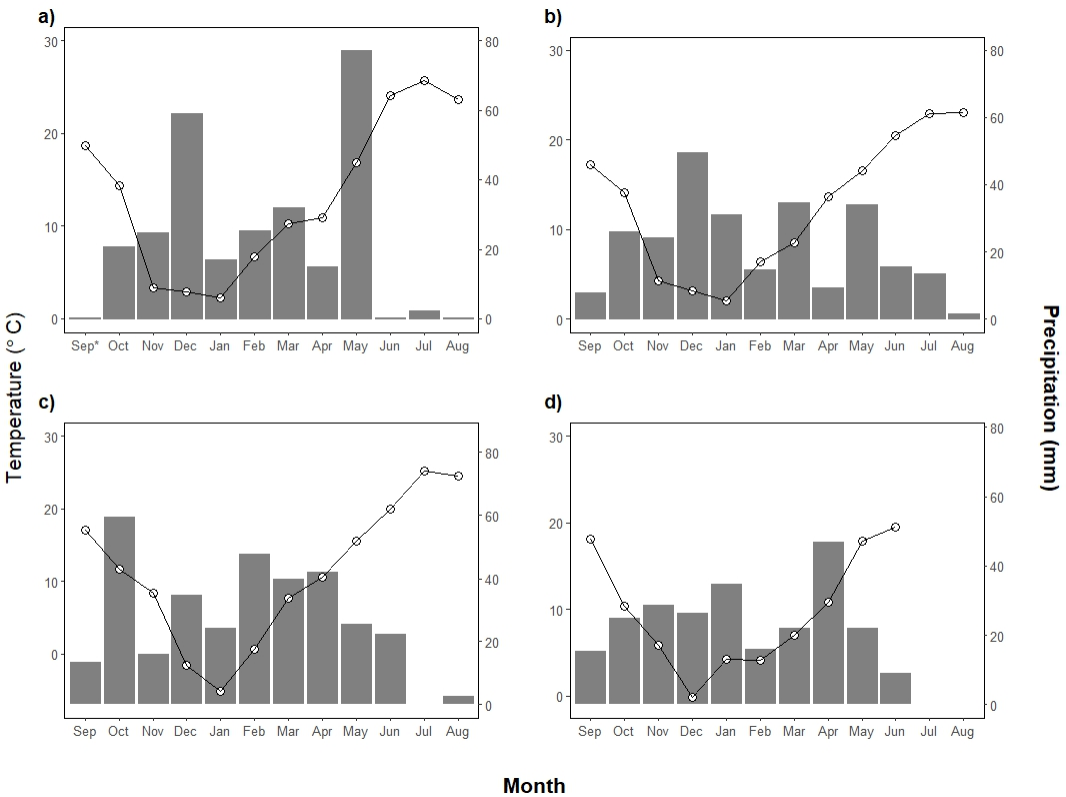
Monthly precipitation and mean temperature at the experimental site for different seasons. **a) 2014–2015, b) 2015–2016, c) 2016–2017, and d) 2017–2018.** The bar graph indicates accumulated rainfalls per month (mm), and the line the average temperature per month (°C). *Precipitation gage was not working properly; this value is a distance-weighted average of nearby Bureau of Reclamation AgriMet Echo site (45.7186^o^ N. Lat., 119.3111 ^o^ W. Lon.) and Citizens Weather Observer Program DW8844 site (45.7255^o^ N. Lat., 118.9250 ^o^ W. Lon.).

The study was undertaken at a 6-ha site near Echo, OR, USA (45.7207^o^ N. Lat., -119.0483^o^ W. Lon.). The soil is a well-drained Ritzville silt loam (coarse-silty, mixed, superactive, mesic Calcidic Haploxeroll) with a surface soil pH of 5.9. Monthly mean temperature was computed from hourly measurements collected at a standard weather station located at the study site (Fig 1). Daily measurements of precipitation were obtained with a tipping bucket rain gauge at the site and used to compile the total precipitation for each month. Mean annual precipitation is 269.4 mm based on long-term records obtained at this site since 2001.

### Experimental design

The experiment was conducted over four growing seasons (2014–2015, 2015–2016, 2016–2017, and 2017–2018). Two cropping systems or rotations, SF/WW and RTF/WW, were established in 2014. The experimental design was a randomized complete block with four replications. Each phase of the rotation (i.e., crop and fallow) was present every season. Therefore, each block had four plots consisting of two WW plots, a fallow plot managed with tillage (SF), and a fallow plot managed with reduced tillage (RTF) bringing the total number of plots to 16 per season. Individual plots were 30.5 m × 6.1 m (100 ft × 20 ft).

Soft white winter wheat (cv. Bobtail in 2015, 2017 and 2018, and cv. Ovation in 2016) was sown into plots with two passes of a 3-m wide Seed Hawk (Langbank, SK, Canada) air till drill with hoe-type openers on 30 cm row spacing. Different crop varieties were used to achieve the highest yield that might be possible for the anticipated conditions as a regular practice in the region (Ovation offers stripe rust tolerance whereas Bobtail offers foot rot resistance). Sowing was performed on 30 November 2014 (delayed due to lack of soil moisture), 13 October 2015, 6 October 2016, and 6 October 2017. Seed density was 132 kg ha^-1^ in 2015, 127 kg ha^-1^ in 2016, 119 kg ha^-1^ in 2017, and 99 kg ha^-1^ in 2018. Fertilizer was applied each year as a solution of 79% urea-ammonium nitrate and 21% ammonium thiosulfate to give rates of 55 kg N ha^-1^ and 11 kg S ha^-1^ (0.794 kg of product in 166.6 l ha^-1^). Herbicide applications (Table 1) were performed with a self-propelled sprayer delivering 187.2 l ha^-1^. Harvest occurred at physiological maturity in mid-July using a small plot combine harvester equipped with 1.9 m header and 9–10 mm top sieve.

**Table 1:** Herbicide applications in the winter wheat plots.

*Year*	*Date*	*Description*
2015	Mar 30	MCPA at 794.8 g ai ha^-1^ + bromoxynil at 741.2 g ai ha^-1^ Thifensulfuron at 12.2 g ai ha^-1^ + tribenuron methyl at 6.1 g ai ha^-1^
	Aug 6	Paraquat dichloride at 3735 g ai ha^-1^
2016	Mar 26	Bromoxynil + pyrasulfotole at 250 g ai ha^-1^ Thifensulfuron at 9.1 g ai ha^-1^ + tribenuron methyl at 9.1 g ai ha^-1^ Mesosulfuron-methyl at 15.6 g ai ha^-1^ Penetrant at 438 g ai ha^-1^ Foam suppressant at 1.8 g ai ha^-1^
	Jul 28	Paraquat dichloride at 2236 g ai ha^-1^
2017	Mar 31	Bromoxynil at 212.1 g ai ha^-1^ + pyrasulfotole at 37.6 g ai ha^-1^ Mesosulfuron-methyl at 15.6 g ai ha^-1^ Penetrant at 467 g ai ha^-1^ Foam suppressant at 11 g ai ha^-1^
	Aug 20	Paraquat dichloride at 2660 g ai ha^-1^ Monocarbamide dihydrogen sulfate at 561 g ai ha^-1^ Penetrant at 701 g ai ha^-1^ Foam suppressant at 13.6 g ai ha^-1^
2018	Apr 9	Bromoxynil at 212.1 g ai ha^-1^ + pyrasulfotole at 37.6 g ai ha^-1^ Mesosulfuron-methyl at 15.6 g ai ha^-1^ Penetrant at 467 g ai ha^-1^ Foam suppressant at 9.1 g ai ha^-1^

Winter wheat plots were subdivided to two small sub-plots: a weedy sub-plot (6.1 m × 3.05 m) where no herbicide was applied and a weed-free sub-plot (6.1 m × 3.05 m) where weeds were pulled by hand. The remaining portion of the plot (6.1 m × 24.4 m) was reserved for general weed management with post-emergence herbicides commonly used in the region.

### Fallow management

The field was in WW in 2012 and it was fallowed in 2013. Starting in 2014, each phase of each rotation was present each year. Winter wheat plots harvested in 2014 completed three cycles of the rotation by 2018 whereas those harvested in 2015 completed two cycles by 2018. Though WW scheduled for harvest in 2015 by design followed fallow, it actually followed two years of fallow because of fallowing of the field in 2013 and presense of a fallow phase in 2014. Weed management in SF consisted of one pass with a disk harrow at a soil depth of 13–15 cm in early spring followed by several passes of a rotary rod weeder in late spring and summer at a depth of 9 cm (Table 2). In RTF, tillage consisted of one pass with an undercutter V-blade sweep in early summer at a depth of 13 cm. Weed control in RTF during spring and summer also included applications of glyphosate (N-(phosphonomethyl) glycine) and other herbicides as needed (Table 2). After harvest, all plots were treated with paraquat (*N*,*N*′-dimethyl-4,4′-bipyridinium dichloride) to control late-season broadleaf weeds that re-grow. Herbicides were applied using a self-propelled sprayer with 6-m spray boom delivering 93.6 l ha^-1^.

**Table 2:** Weed management in the fallow year of the two cropping systems.

Summer fallow			Reduced tillage fallow		
*Year*	*Date*	*Description*	*Year*	*Date*	*Description*
2013	Oct 24	Glyphosate application at 3081 g ai ha^-1^	2013	Oct 24	Glyphosate application at 3081 g ai ha^-1^
2014	Mar 22	Tillage with a disk harrow	2014	Apr 29	Glyphosate application at 1549 g/ha
	May 22	Tillage with a rod weeder		May 29	Tillage with a sweep cultivator
	Aug 21	Paraquat dichloride application at 2218 g ai ha^-1^		Aug 21	Paraquat dichloride application at 2218 g ai ha^-1^
2015	Feb 17	Glyphosate application at 1684 g ai ha^-1^	2015	Feb 17	Glyphosate application at 1684 g ai ha^-1^
	Apr 21	Tillage with a disk harrow		Apr 30	Glyphosate application at 3081 g ai ha^-1^
	Jun 12	Tillage with a rod weeder		Jun 15	Glyphosate application at 3081 g ai ha^-1^
				Jun 23	Tillage with a sweep cultivator
	Aug 6	Paraquat dichloride application at 3735 g ai ha^-1^		Aug 6	Paraquat dichloride application at 3735 g ai ha^-1^
2016	Feb 26	Glyphosate application at 3081 g ai ha^-1^, monocarbamide dihydrogen sulfate at 187 g ai ha^-1^ and, foam suppressant at 7.3 g ai ha^-1^	2016	Feb 26	Glyphosate application at 3081 g ai ha^-1^, monocarbamide dihydrogen sulfate at 187 g ai ha^-1^ and, foam suppressant at 7.3 g ai ha^-1^
	Mar 29	Tillage with a disk harrow		May 17	Glyphosate application at 3081 g ai ha^-1^, thifensulfuron at 9.1 g ai ha^-1^+ tribenuron methyl at 9.1 g ai ha^-1^, penetrant at 467 g ai ha^-1^ and, foam suppressant at 5.5 g ai ha^-1^
	May 25	Tillage with a rod weeder		Jul11	Tillage with a sweep cultivator
	Jul 27	Tillage with a rod weeder			
	Jul 28	Paraquat dichloride application at 2236 g ai ha^-1^		Jul 28	Paraquat dichloride application at 2236 g ai ha^-1^
2017	Mar 19	Glyphosate application at 1549 g ai ha^-1^, water conditioning agent at 233 g ai ha^-1^, penetrant at 467 g ai ha^-1^ and, foam suppressant at 7.3 g ai ha^-1^		Mar 19	Glyphosate application at 1549g ai ha^-1^, water conditioning agent at 233 g ai ha^-1^, penetrant at 467 g ai ha^-1^ and, foam suppressant at 7.3 g ai ha^-1^
	May 2	Tillage with a disk harrow		May 23	Glyphosate application at 1549 g ai ha^-1^, dicamba at 414 g ai ha^-1^, monocarbamide dihydrogen sulfate at 374 g ai ha^-1^, penetrant at 467 g ai ha^-1^ and, foam suppressant at 9.1 g ai ha^-1^
	May 10	Tillage with a rod weeder		Jul 3	Tillage with a sweep cultivator
	Jun 22	Tillage with a rod weeder			
	Aug 20	Paraquat dichloride application at 2660 g ai ha^-1^, monocarbamide dihydrogen sulfate at 561 g ai ha^-1^, penetrant at 701 g ai ha^-1^ and, foam suppressant at 13.6 g ai ha^-1^		Aug 20	Paraquat dichloride application at 2660 g ai ha^-1^, monocarbamide dihydrogen sulfate at 561 g ai ha^-1^, penetrant at 701 g ai ha^-1^ and, foam suppressant at 13.6 g ai ha^-1^
2018	Oct 5	Tillage with a rod weeder		Oct 4	Glyphosate application at 1157 g ai ha^-1^, water conditioning agent at 233 g ai ha^-1^, penetrant at 467 g ai ha^-1^ and, foam suppressant at 9.1 g ai ha^-1^
	Feb 13	Glyphosate application at 1549 g ai ha^-1^, monocarbamide dihydrogen sulfate at 374 g ai ha^-1^, penetrant at 467 g ai ha^-1^ and, foam suppressant at 7.3 g ai ha^-1^		Feb 13	Glyphosate application at 1549 g ai ha^-1^, monocarbamide dihydrogen sulfate at 374 g ai ha^-1^, penetrant at 467 g ai ha^-1^ and, foam suppressant at 7.3 g ai ha^-1^
	Apr 2	Tillage with a disk harrow		May 2	Glyphosate application at 1549 g ai ha^-1^, monocarbamide dihydrogen sulfate at 187 g ai ha^-1^, penetrant at 467 g ai ha^-1^ and, foam suppressant at 4.6 g ai ha^-1^
	May 23	Tillage with a rod weeder		Jun 15	Glyphosate application at 1924 g ai ha^-1^, glyphosate at 508.9 g ai ha^-1^ + 2,4-D at 812.6 g ai ha^-1^, monocarbamide dihydrogen sulfate at 374 g ai ha^-1^, penetrant at 234 g ai ha^-1^ and, foam suppressant at 0.5 g ai ha^-1^
	Jul 16	Tillage with a rod weeder		Jul 16	Tillage with a sweep cultivator

### Data collection

Weed density and cover were measured within 16 sampling frames (1 m × 0.5 m) placed in each WW plot: four in the weedy sub-plot, four in the weed-free sub-plot, and eight in the general area. Sampling frames were constructed from 1.25 cm diameter PVC pipe. Winter wheat plots were sampled three times during the growing season: early season at the beginning of weed competition (mid-March, tillering), mid-season at peak crop growth (end of May, flowering), and late-season at crop maturity (end of June, near harvest). At each sampling time, percent cover of the crop and each weed species were estimated visually within each frame. Density (plants m^-2^) of the crop and each weed species were determined by counting the number of plants in each frame. At harvest (i.e., late season sampling), the standing wheat in each frame was collected by destructive sampling and bundled. Bundles of wheat were then threshed, and the resulting grain was cleaned and weighed for determination of yield.

### Statistical analysis

A generalized linear mixed model (GLMM) was fit to test for differences between SF and RTF in terms of density and cover of grass weeds, broadleaf weeds, and total weeds. Models were fit with different data distributions by year and plot area (weedy vs. general) because weed pressure significantly differed among years and between plot areas each growing season. Mixed models allowed for incorporation of different levels of pseudo-replication inherent to plot sampling. Type of fallow management, crop density or crop cover, and their interactions were entered as fixed effects. Crop density and cover were centered around their mean. Plot was included as a random effect. In all models, assumptions of equal variances and normal distribution of residuals were evaluated graphically. These analyses were implemented with thelme4 [41] package in the R program v. 3.3.2 [42].

Non-linear regression was used to test the effect of weeds on wheat yield in relation to fallow management. The non-linear models were the hyperbolic rectangular [Eq 1] [43,44] and negative exponential [Eq 2] [45,46]:

$$Y=Y_{o}(1-\frac{aX}{\left(1+\frac{aX}{b}\right)})$$ [Eq 1]

$$Y=Y_{o}e^{-cX}$$ [Eq 2]

where *Y* is yield in presence of weeds (kg ha^-1^), *Y*_*o*_ is yield in absence of weeds (kg ha^-1^), *X* is either weed density (plants m^-2^) or weed cover (%), *a* is yield loss per *X* unit as weed presence approaches zero, *b* is yield loss as *X* approaches infinity, and *c* estimates the rate of reduction in crop yield as weed density or cover increases. Models were selected based on the corrected Akaike information criterion (AICc). The non-linear mixed model (NLMM) analysis was used to identify differences between model parameters (*Y*_*o*_ and *c*). These parameters were included as fixed effects and plot as a random effect. These analyses were implemented with the nlme [47] package in the R program [42].

Those frames in which a species is present vs. total number of frames was calculated to determine percent species presence in each year. Non-parametric multivariate analysis of variance (PERMANOVA) was conducted with “adonis” function available in vegan package [48] of the R program [42] to search for significant differences between the weed community of the two cropping systems. Type of fallow management, replicate, and their interaction were used as factors.

### Cost comparison

The total U.S. dollar cost of each fallow management was estimated by year, by summing the individual costs of the different management operations (Table 2). Cost of various mechanical and chemical operations in fallow/WW production systems of the area are quoted in Seavert et al. [49] at $11.10 ha^-1^ for herbicide application, $24.70 ha^-1^ for disk harrowing, $14.80 ha^-1^ for rod weeding, and $14.80 ha^-1^ for sweep cultivation. Each herbicide application cost per hectare was calculated by multiplying the application rate times the retail prices for individual herbicides listed online at the University of Nebraska [50]. For example, if application rate is 4.67 L ha^-1^ (3081 g ai ha^-1^) and retail price of Roundup Power Max^®^ is $6.60 L^-1^ (660 g a.i.), then herbicide application cost is $30.80 ha^-1^.

## Results and discussion

### Weed composition and year effect

Total precipitation during each cropping year (September to August) was 273 mm (101% of long-term average) in 2015, 262 mm (97%) in 2016, 327 mm (121%) in 2017, and 246.2 mm (92%) in 2018. Mean density (plants m^-2^) of total weeds in weedy sub-plots increased from 7.9 ± 5.8 in 2015 to 25.4 ± 24.0 in 2016, 51.6 ± 32.4 in 2017, and 88.0 ± 44.2 in 2018. Mean cover (%) of total weeds also increased with year from 21.0 ± 23.4 in 2015 to 26.1 ± 28.8 in 2016, 79.0 ± 44.7 in 2017, and 75.8 ± 33.3 in 2018. Dominant weeds were tumble mustard, downy brome, prickly lettuce, Russian thistle, and volunteer wheat as indicated by percentage of presence (Table 3). In eastern Washington, Schillinger [1] found downy brome and Russian thistle to be the most dominant species whereas tumble mustard had a minor presence. These weed species were influenced by growing conditions. Other studies have found that the amount and distribution of precipitation strongly influenced the seed germination [51]. In this study, downy brome, tumble mustard and prickly lettuce increased over time while Russian thistle and kochia decreased. More species appeared during our study (*e*.*g*., coast fiddleneck, flixweed, panicle willow weed and horseweed) than disappeared (*e*.*g*., lambsquarters). Significant differences in weed community composition between SF/WW and RTF/WW were not found by multivariate analyses.

**Table 3:** Percentage of presence of all species in the weedy (W) and general (G) area, for all samplings in the different years.

			Year (% presence^b^)				
Scientific name	Code^a^	Common name	2015	2016	2017	2018	Average
***Winter annuals***							
*Sisymbrium altissimum* L.	SSYAL	Tumble mustard	39.6	54.9	55.6	65.1	53.8
*Bromus tectorum* L.	BROTE	Downy brome	11.1	24.3	74.3	77.1	46.7
*Lactuca serriola* L.	LACSE	Prickly lettuce	3.8	19.8	42.1	59.9	31.4
*Triticum aestivum* L.	TRAE	Volunteer wheat	4.5	17.4	10.1	24.5	14.1
*Avena fatua* L.	AVEFA	Wild oat	1.4	21.2	0.7	1.6	6.2
*Brassica napus L*.	BRSNN	Volunteer canola	0.0	3.1	5.6	3.7	3.1
*Secale cereal* L.	SECE	Cereal rye	0.0	1.0	2.8	1.6	1.4
*Amsinckia menziesii* (Lehm.) A. Nels. & J.F. Macbr. var. *intermedia*	AMSIN	Coast fiddleneck	0.0	0.0	1.7	0.0	0.4
*Descurainia sophia* (L.) Webb. ex Prantl	DESSO	Flixweed	0.0	0.4	1.4	0.0	0.5
*× Triticosecale* Wittm. ex A. Camus.	-	Triticale	0.0	0.4	1.0	2.6	1.0
*Lamium amplexicaule* L.	LAMAM	Henbit	0.0	0.4	0.4	0.0	0.2
***Summer annuals***							
*Salsola tragus* L.	SASKR	Russian thistle	24.7	16.7	2.8	2.1	11.6
*Epilobium brachycarpum* K. Presl	EPIPC	Panicle willowweed	0.00	0.4	13.9	14.1	7.1
*Kochia scoparia* (L.) Schrad.	KCHSC	Kochia	5.6	2.4	1.4	1.6	2.8
*Chenopodium leptophyllum* (Moq.) Nutt. ex S.Wats	CHELE	Lambsquarters	1.0	0.7	0.0	0.0	0.4
*Amaranthus* sp.	AMA	Pigweed	0.0	0.4	1.0	0.0	0.4
*Polygonum aviculare* L	POLAV	Prostrate knotweed	0.0	0.7	0.0	0.5	0.3
*Conyza canadensis* (L.) Cronq.	ERICA	Horseweed	0.0	0.0	0.4	1.6	0.5
***Biennials***							
*Tragopogon dubius* Scop.	TRODM	Western salsify	1.0	0.0	1.4	1.6	1.0

^a^Code and common name based on Weed Science Society of America (WSSA) classification (Bayer Code).

^b^Percentage of presence is referred to number of frames relative to the total number of frames in which a species is present.

Grass weed infestation was lower in 2015 than 2016, 2017 and 2018 apparently due to seedbank depletion over two years of fallow in which a single application of glyphosate was applied each year. Downy brome increased the most from the beginning of the study in terms of species presence (percentage of frames in which a species is present vs. total number of frames) and abundance (plants m^-2^ and/or percentage cover). Downy brome abundance may have increased because of the absence of a post-emergence herbicide application on WW plots in 2015 due to the low density of this species and other grass weeds in that year (0.56 plants m^-1^). However, increased precipitation, especially in 2017, likely enhanced conditions for germination and development of downy brome resulting in its increased presence and abundance in that year. Due to the great proliferation of downy brome in 2017, the seedbank could have increased, and consequently led to a great abundance in the following growing season in 2018. Schillinger [1] and Blackshaw et al. [51] found that favorable moisture conditions increased density of downy brome and flixweed. Flixweed is a species with a similar biology to tumble mustard that also increased in our study under those conditions. In contrast, Russian thistle infestation decreased over the three years of this study. Drier weather conditions and the absence of other weeds may have favored this species in 2015. Russian thistle was found to have a low tolerance to competition, growing better in crop- and weed-free sub-plots [52] as well as after harvest. Schillinger [1] also reported scarcity of this species in humid years, as did Blackshaw et al. [51] who found more density of this species in low rainfall periods. Year 2015 was the warmest of the four growing seasons, which could explain the greatest Russian thistle presence in that year along with the late spring rainfall (Fig 1) that could have also caused new flushes.

### Effect of fallow management on following season’s weeds

**Fig 2:**
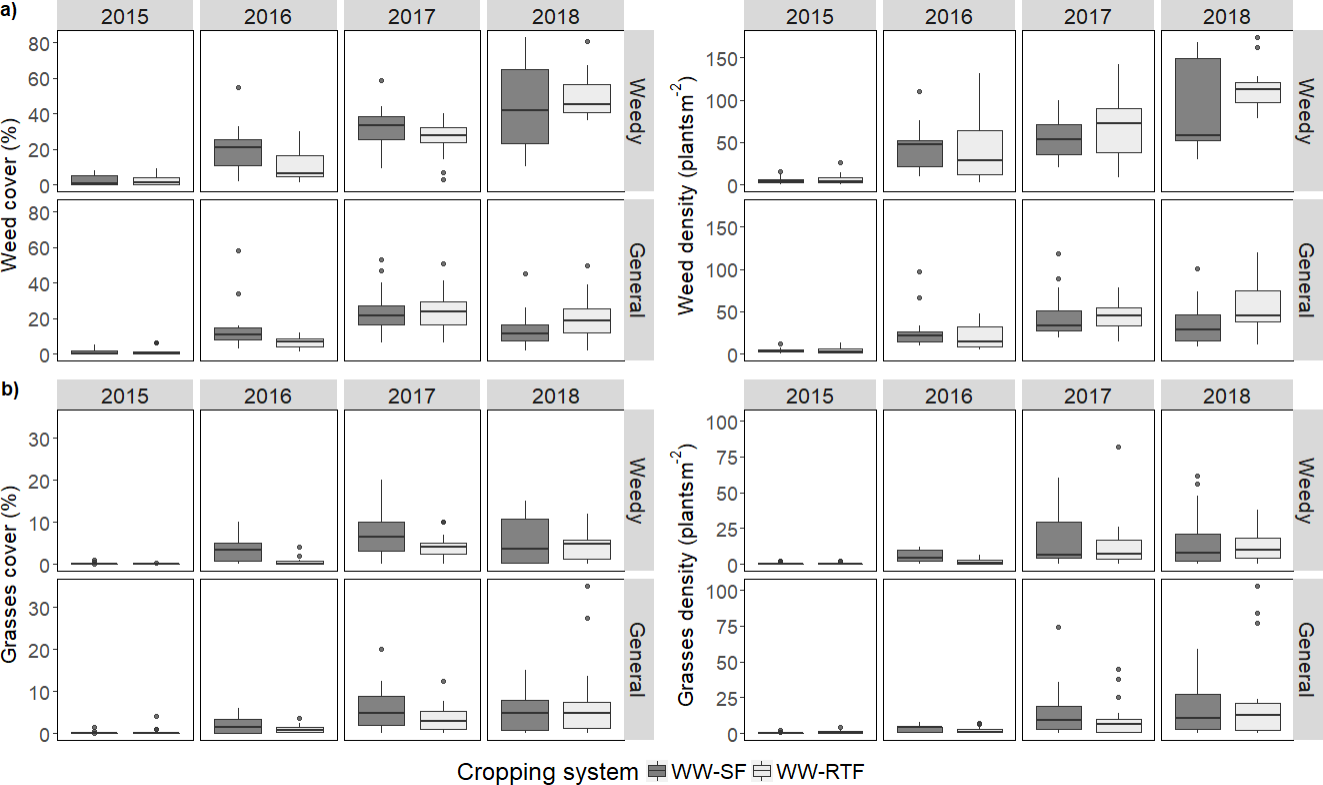
Total weed density and cover (a) and grass weed density and cover (b) observed in the weedy and general areas in the cropping systems (Winter wheat (WW)–Summer Fallow (SF) and WW–Reduced Tillage Fallow (RTF)) along the study (2015–2018).

For total weeds and broadleaf weeds, differences in density were not detected between fallow managements, except in 2018, where higher broadleaf weed density was obtained in the WW plots managed with RTF. The percentage cover of broadleaf and total weeds was reduced in the WW with RTF compared to SF in 2016 (Table 4, Fig 2), was not affected with fallow management in 2016 and 2017, and increased in 2018 in the RTF compared to SF, although only in the general area. Broadleaf weeds contributed the most to the total weed density (90.3% in 2015, 90.2% in 2016, 72.4% in 2017, and 70.3% in 2018) and cover (94.9% in 2015, 87.0% in 2016, 80.7% in 2017, and 78.3% in 2018). Tumble mustard contributed the most to total broadleaf weed cover and density (56.1% in 2015, 82.8% in 2016, 80.3% in 2017, and 84.9% in 2018). Evans and Young [53] found emergence of tumble mustard was favored when the soil surface is rough and pitted. Though the soil surface in RTF was rougher and with more pits than in SF, tumble mustard density was not significantly different between fallow managements in our study most of the years except for 2018. Prickly lettuce and volunteer wheat did not significantly differ in cover and density between fallow managements (data not shown). Differences in Russian thistle density and cover could not be detected between SF and RTF in 2015 and 2016 (its presence was almost zero in 2017 and 2018). Young and Thorne [3] found that Russian thistle was stimulated by tillage. However, Schillinger [1, 54] did not find differences between tillage regimes and rotations for that species.

**Table 4:** Total weed, broadleaved, and grass weed cover and density by year as affected by fallow management and crop cover. Note: analysis for grasses in 2015 was not included because grasses were almost absent in 2015.

		2015		2016		2017		2018	
Response		Estimate	p-value ^a^	Estimate	p-value	Estimate	p-value	Estimate	p-value
		*Weedy sub-plots*								
**Total weed cover ^**d**^**	(Intercept) ^b^	0.558	0.366	2.967	<0.001***	33.22	<0.001***	43.62	<0.001***
	Management ^c^	-0.392	0.639	-0.924	0.003**	-7.23	0.209	6.411	0.505
	Crop cover	-0.088	0.526	-0.007	0.336	0.492	0.110	-0.686	0.045*
	Management×Crop cover	-0.007	0.965	0.054	<0.001***	-0.543	0.240	0.132	0.789
**Total weed density ^**d**^**	(Intercept)	1.538	<0.001***	3.746	<0.001***	55.74	<0.001***	86.15	<0.001***
	Management	0.051	0.898	-0.327	0.486	11.51	0.576	24.27	0.336
	Crop density	0.011	0.175	0.001	0.044*	0.154	0.088.	-0.111	0.604
	Management×Crop density	-0.010	0.280	-0.003	<0.001***	-0.065	0.627	0.306	0.302
**Broadleaved weed cover ^**d**^**	(Intercept) ^b^	1.757	0.699	3.272	<0.001***	19.10	0.006**	46.88	<0.001***
	Management ^c^	0.802	0.866	-2.911	<0.001***	3.821	0.714	14.80	0.357
	Crop cover	-0.038	0.819	-0.015	**0.067.**	0.295	0.299	-0.351	0.250
	Management×Crop cover	-0.040	0.821	0.062	<0.001***	-0.342	0.424	-0.269	0.573 3
**Broadleaved weed density ^**d**^**	(Intercept)	0.271	0.780	3.405	<0.001***	24.58	<0.001***	4.021	<0.001***
	Management	1.398	0.261	0.173	0.745	9.689	0.386	0.395	**0.095.**
	Crop density	0.010	0.195	0.001	0.210	0.130	<0.001***	0.002	0.022*
	Management×Crop density	-0.011	0.247	-0.002	<0.001***	0.034	0.785	-0.000	0.876
**Grass weed cover**	(Intercept)			1.230	<0.001***	1.915	<0.001***	1.253	<0.001***
	Management			-1.935	<0.001***	-0.574	**0.085.**	0.004	0.994
	Crop cover			0.015	0.596	0.017	0.125	-0.032	**0.019** *
	Management×Crop cover			-0.009	0.879	-0.023	0.308	0.032	0.128
**Grass weed density**	(Intercept)			1.740	<0.001***	2.354	<0.001***	2.271	<0.001***
	Management			-1.249	<0.001***	-0.120	0.861	0.212	0.626
	Crop density			0.000	0.681	0.001	0.574	-0.011	<0.001***
	Management×Crop density			-0.002	0.683	-0.006	<0.001***	0.014	<0.001***
		*General area*							
**Total weed cover**	(Intercept)	-0.347	0.491	2.680	<0.001***	21.96	0.004**	2.483	<0.001***
	Management	0.662	0.305	-0.881	<0.001***	3.570	0.657	0.687	0.049*
	Crop cover	0.195	0.199	0.015	**0.061.**	-0.652	0.390	-0.000	0.931
	Management×Crop cover	-0.209	0.226	-0.014	0.351	0.424	0.617	-0.045	0.001**
**Total weed density**	(Intercept)	1.393	<0.001***	3.188	<0.001***	41.61	<0.001***	3.550	<0.001***
	Management	-0.292	0.527	-0.264	0.375	0.613	0.965	0.363	0.376
	Crop density	-0.001	0.906	-0.001	0.155	-0.108	0.448	0.004	<0.001***
	Management×Crop density	0.005	0.720	0.001	0.100	0.182	0.256	-0.003	0.030*
**Broadleaved weed cover ^**d**^**	(Intercept) ^b^	-0.635	0.308	2.528	<0.001***	2.845	<0.001***	1.914	<0.001***
	Management ^c^	0.574	0.463	-0.947	<0.001***	0.206	0.476	0.749	**0.073.**
	Crop cover	0.288	0.166	0.017	0.049*	0.006	0.724	-0.009	0.375
	Management×Crop cover	-0.208	0.371	-0.007	0.676	-0.022	0.234	-0.024	0.121
**Broadleaved weed density ^**d**^**	(Intercept)	1.320	<0.001***	3.041	<0.001***	3.240	<0.001***	2.731	<0.001***
	Management	-0.510	0.323	-0.264	0.435	0.235	0.415	0.625	0.024*
	Crop density	0.001	0.926	-0.001	0.188	-0.001	0.378	-0.003	0.049*
	Management×Crop density	0.003	0.808	0.002	**0.050.**	0.001	0.556	0.005	0.011*
**Grass weed cover**	(Intercept)			0.707	0.01**	0.603	0.221	1.358	<0.001***
	Management			-0.785	**0.083.**	0.231	0.732	0.563	0.377
	Crop cover			0.034	0.188	-0.223	<0.001***	0.019	0.323
	Management×Crop cover			-0.105	**0.058.**	0.254	<0.001***	-0.099	<0.001***
**Grass weed density**	(Intercept)			1.172	<0.001***	2.297	<0.001***	2.622	<0.001***
	Management			-0.393	0.255	-0.783	0.287	0.132	0.870
	Crop density			-0.000	0.962	-0.006	<0.001***	0.011	<0.001***
	Management×Crop density			-0.001	0.687	0.016	<0.001***	-0.011	<0.001***

^a^Significance codes for p-values obtained after the GLMM: p<0.1

* p<0.05

**p<0.01

***p<0.001

^b^Intercept is the expected mean value of dependent variable when all predictor variables = 0. Due to we have centered the crop cover and crop density variables around their mean values, the intercept only refers to Management (SF).

^c^Management is referred to Reduced Tillage Fallow (RTF).

^d^Broadleaf weeds density and cover had the same significant values and the same estimate sign than the total weeds.

The paucity of weeds in 2015 prevented statistical comparison of grass weed cover between weedy sub-plots of SF and RTF in that year. In contrast, grass weed cover of weedy sub-plots was significantly higher in SF than RTF in 2016 and 2017 (Table 4, Fig 2). The difference in grass weed cover in 2016 may be related to reduced grass weed density in RTF versus SF that year, though a significant difference in grass weed density was not found in 2017. Sampling of weeds was performed at the same time in SF and RTF. Consequently, less grass weed cover at a similar density implies delay in emergence, development, or both in WW following RTF. Therefore, untreated weedy sub-plots had grass infestations that were more competitive in WW plots preceded by SF than WW plots preceded by RTF. In general areas, having a small seedbank due to weed control every year, differences of marginal significance (P < 0.1) were only found in 2016 (Table 4).

Downy brome contributed the most to total grass weed density (70.4% in 2015, 52.5% in 2016, 94.3% in 2017, and 92.4% in 2018). Previous workers reported that emergence of downy brome is favored by uncompacted soils [55,56] in agreement with this study. Summer fallowing by means of disk harrowing and rod weeding creates a finely textured layer of soil with reduced compaction compared to RTF involving a single V-blade sweep operation. Thorne et al. [39] and Kettler et al. [40] found downy brome control improved in no-till cropping systems with occasional tillage. In this study, reducing tillage from two to three operations per season (SF) to one tillage operation per season (RTF) reduced downy brome the following season.

Moreover, leaving higher amounts of residue on the soil surface are known to increase the suppression of weeds [28–30]. Reduced tillage fallow resulted in more crop residue cover and surface roughness than SF, which could explain the higher weed cover found in WW following the SF. Residue suppresses or delays weed germination and emergence by reducing soil temperature [57] and light [29], emitting allelopathic substances [58], and increasing competition for free-space [29,59] but the contribution of each factor to the weed suppression is difficult to determine [59].

However, seed germination inhibited by light exposure (negative photoblastism) has been determined in *Bromus* species [60]. Del Monte and Dorado [61] found that ripgut brome (*Bromus diandrus* Roth) seeds buried or shaded superficially can germinate during fall. Recasens et al. [62] found that germination of ripgut brome was favored by chisel plowing compared to moldboard plowing that buried seeds deeper. Intensive tillage in the SF apparently may have buried seeds and limited their exposure to light, which enhanced germination of downy brome in the following crop. Though the shading effect of crop residue might be greater than seed burial, it can suppress downy brome germination and emergence because it works as a physical barrier that can help with weed control. In RTF, the amount of remained crop residue before seeding is larger than in SF and might have contributed to reduce weed development in the WW. However, non-significant differences in weed infestation between fallow managements in 2017, except for the grass cover in the weedy sub-plots, indicates that when the soil moisture is abundant, the effect of residue on weed infestations could be overcome, especially for broadleaf weeds. The same situation could have happened with grasses in 2018 where no differences were found in relation to density and cover for both weedy and general areas (Table 4, Fig 2). In 2018, unusually warm January temperatures coupled with adequate precipitation (Fig 1) might have favored higher, later downy brome germination than in previous years such that fallow management was ineffective and unnoticeable in a well-established crop.

### Crop yield in relation to fallow management and weed infestation

Overall, WW yield in the general area was greater in 2015 (3011 kg ha^-1^) and 2017 (3554 kg ha^-1^) than in 2016 (1854 kg ha^-1^) and 2018 (1977 kg ha^-1^) regardless of fallow management. In 2015, crop yield was not significantly impacted by weed presence due to low weed infestation (5.5 plants m^-2^). Despite similar amounts of precipitation, 2015 yields were higher than 2016 yields likely due to soil water storage over two years of preceding fallow but also higher May precipitation (Fig 1A). Mean weed infestation in the general area fell from 45 plants m^-2^ at the beginning of growing season to 11 plants m^-2^ at harvest. Infestations were not correlated with crop yield in the general area (data not shown). Yield was not impacted by initial weed infestation indicating that weeds were adequately controlled with herbicides.

**Fig 3:**
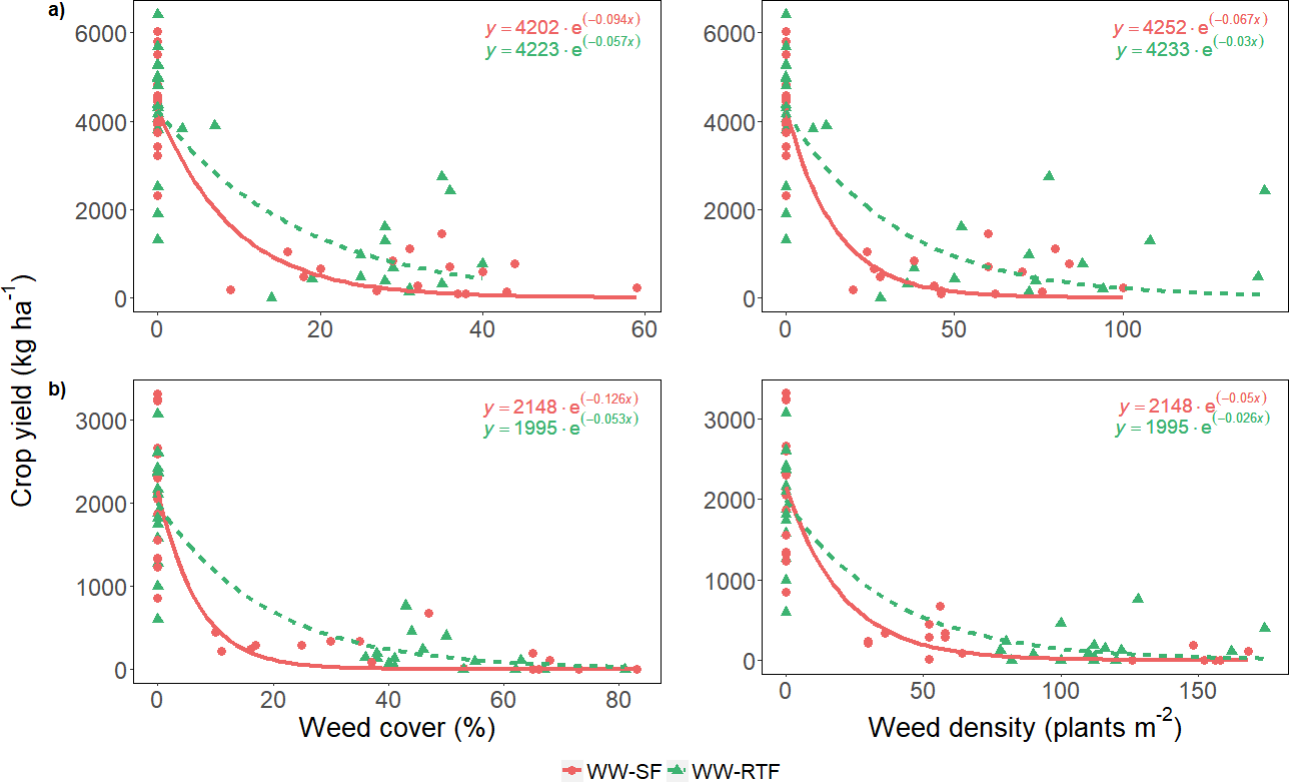
Relationship between crop yield (kg ha^-1^) and weed density (plants m^-2^) and weed cover (%) in 2017 (a) and 2018 (b).

Mean grain yield in the weedy sub-plots of WW plots was significantly greater in 2015 (2858 kg ha^-1^) than in 2016 (1360 kg ha^-1^), 2017 (904 kg ha^-1^) and 2018 (188 kg ha^-1^). Yields of weedy sub-plots were only 25.4% and 9.5% of the general area due to high average weed densities of 52 and 88 plants m^-2^ in 2017 and 2018. From 2016 to 2018, with higher weed pressure than in 2015, grain yield decreased with increasing weed density or cover in the weedy sub-plots (Fig 3). The exponential model (Fig 3) was chosen to represent the relationship between crop yield and weed infestation because it had lower AICc than the hyperbolic model. Parameters *Y*_*0*_ and *c* in the selected models were non-significant for fallow managements in all years (Table 5). Nevertheless, the curvature of the fit *(c)* representing the rate of change in grain yield for weed density and weed cover was lower in RTF than in SF, especially for 2017 and 2018, indicating that the negative effect of weeds on crop yield was greater in SF than in RTF. Differences in crop yield between fallow managements were smaller in 2016 than in 2017 and 2018 (Table 5). Crop yield did not differ between fallow management when weed presence was zero (i.e., *Y*_*0*_) whereas the same weed infestations caused larger yield loss in cropping systems with SF than in cropping systems with RTF.

**Table 5:** Non-linear mixed model (NLMM) analysis to study the parameters significance of the relationship (represented by the exponential model) between crop yield and weed cover, and crop yield and weed density in the two fallow managements (summer fallow (SF) and reduced tillage fallow (RTF)).

		2016		2017		2018	
Models	Parameters	Estimate	p-value^a^	Estimate	p-value	Estimate	p-value
**Crop yield in response to weed cover**	*Y*_*0*_: Intercept ^b^	1884.7	0.000***	4201.7	0.000***	2147.8	0.000***
	*ΔY*_*0*_: Management (RTF)	57.07	0.506	21.50	0.965	-152.3	0.626
	*c*: Intercept ^c^	0.024	0.001**	0.094	.002**	0.126	0.008**
	*Δc*: Management (RTF)	-0.002	0.449	-0.037	0.243	-0.073	0.134
**Crop yield in response to weed density**	*Y*_*0*_: Intercept	1882.9	0.000***	4252.1	0.000***	2147.9	0.000***
	*ΔY*_*0*_: Management (RTF)	103.1	0.500	-19.40	0.966	-153.2	0.621
	*c*: Intercept	0.010	0.001**	0.067	0.006**	0.050	0.000***
	*Δc*: Management (RTF)	-0.003	0.561	-0.037	0.138	-0.024	0.152

^a^Significance codes for p-values obtained after the NLMM: p<0.1

* p<0.05

**p<0.01

***p<0.001

^b^Intercept *Y*_*0*_ is the expected mean value of SF and ΔY_0_ is the difference in mean yield between SF and RTF.

^c^Intercept *c* is the expected mean value of SF and Δ*c* is the difference in mean yield between SF and RTF.

Weeds were less competitive in RTF in 2016, 2017 and 2018 resulting in higher mean crop density and cover in the WW following RTF than in the WW following SF in those years. In 2016, the WW following RTF had 200.8 wheat plants m^-2^ and 35.0% cover compared to 135.3 wheat plants m^-2^ and 27.1% cover for the WW following SF. In 2017, weed density and cover were 135.8 plants m^-2^ and 23.0% for WW following RTF, and 84.0 plants m^-2^ and 16.9% for WW following SF. However, differences were not significant for crop cover in 2017. And in 2018, weed density and cover were 102.8 plants m^-2^ and 29.1% for WW following RTF, and 60.2 plants m^-2^ and 21.6% for WW following SF.

### Implications for management and concluding remarks

In this study, fallow management influenced weed cover and density in WW, with RTF resulting in the development of smaller plants early in the season and improving suppression of downy brome over that of SF. However, environmental conditions could diminish the effect of RTF as in January 2018 when warm and wet conditions promoted late downy brome germination. Young and Schillinger [63] reported that farmers in the PNW used nearly equivalent amounts of glyphosate and obtained similar profitability under SF and RTF. In this study, weed management costs were similar between SF and RTF in 2016 ($157 vs. $164 ha^-1^) and 2017 ($181 vs. $195 ha^-1^) but were larger for RTF in 2015 ($130 vs. $189 ha^-1^) and 2018 ($107 vs. $175 ha^-1^). Adjuvants are normally used in commercial fields but in this study they were required in higher amounts to prevent herbicide drift between narrow plots. Therefore, costs obtained for RTF should be considered illustrative. Chemical costs could also be reduced in RTF by rotating herbicides instead of relying upon tank mixtures.

The question remains whether greater use of herbicides in RTF negatively impacts the environment and human health. Reduction in herbicide use might be achieved after several seasons given that the first years after the adoption of conservation tillage, herbicides play an important role in controlling weeds [18]. Sebastian et al. [64] found the seedbank of downy brome became depleted after 4–5 consecutive years of glyphosate applications. A reduction in herbicide application could also be achieved by diversifying the fallow / WW rotation with crops that can break pest cycles. Finally, long-term approaches to integrated weed management should reduce reliance upon herbicides [65] and the extent of herbicide-resistant species [66].

## Supporting information

S1 File Final dataset. (CSV)
